# Factors Associated With the Severity of Chronic Constipation Among Japanese University Students

**DOI:** 10.1002/jgh3.70218

**Published:** 2025-07-22

**Authors:** Nhu Thi Hanh Vu, Huong Tu Lam, Shunsuke Miyauchi, Naoki Ishiuchi, Atsuo Yoshino, Yoshie Miyake, Shiro Oka, Yuri Okamoto, Duc Trong Quach, Shinji Tanaka, Toru Hiyama

**Affiliations:** ^1^ Department of Internal Medicine School of Medicine ‐ University of Medicine and Pharmacy at Ho Chi Minh City Ho Chi Minh Vietnam; ^2^ Department of Endoscopy Hiroshima University Hospital Hiroshima Japan; ^3^ Health Service Center, Hiroshima University Higashihiroshima Japan; ^4^ Department of Gastroenterology Graduate School of Biomedical and Health Sciences, Hiroshima University Hiroshima Japan; ^5^ JA Onomichi General Hospital Onomichi Japan

**Keywords:** chronic constipation, lifestyle factors, moderate‐to‐severe constipation, psychological assessment

## Abstract

**Background and Aim:**

Chronic constipation (CC) is a prevalent gastrointestinal disorder in which individuals with more severe CC significantly decrease their quality of life and often require more active medical intervention. This study aimed to identify the rate and risk factors associated with moderate‐to‐severe CC among Japanese university students.

**Methods:**

A cross‐sectional study was conducted among university students diagnosed with CC according to the Rome IV criteria at Hiroshima University, Hiroshima, Japan. The online questionnaire collected data on demographics, lifestyle habits, family history of constipation, and psychological assessments via the Beck Depression Inventory, Eating Attitudes Test‐26, and Bulimic Investigatory Test. CC severity was classified based on the Bristol Stool Form Scale (BSFS), spontaneous bowel movements (SBM), and associated symptoms. Multivariate logistic regression analysis was performed to determine independent risk factors.

**Results:**

Among the 779 CC participants, 47% were classified as having moderate‐to‐severe CC. Independent risk factors associated with moderate‐to‐severe CC included age of CC onset < 18 years (OR: 1.374, 95% CI: 1.017–1.857), female sex (OR: 1.444, 95% CI: 1.058–1.969), family history of CC (OR: 1.449, 95% CI: 1.072–1.958), and sleep duration ≤ 6 h per day (OR: 1.350, 95% CI: 1.011–1.802).

**Conclusions:**

Moderate‐to‐severe CC was highly common among Japanese students with CC, and risk factors included early onset, female sex, family history of CC, and short sleep duration. These findings suggest that early diagnosis and development of management strategies may be needed to enhance the quality of life and health outcomes of affected individuals.

**Abbreviations**
BDI: Beck Depression Inventory scale
BITE: Bulimic Investigatory Test
BSFS: Bristol Stool Form Scale
CC: chronic constipation
CI: confidence interval
EAT: Eating Attitudes Test
OR: odds ratio
SBM: spontaneous bowel movements

## 1 Introduction

Chronic constipation (CC) is one of the most prevalent gastrointestinal disorders, affecting approximately 12%–19% of the global population [1, 2, 3, 4]. CC has a substantial negative impact on both physical and emotional well‐being, leading to poor health consequences, reduced quality of life, and impaired work productivity and school attendance [5, 6, 7, 8]. In addition, individuals with CC incur increased healthcare utilization and financial costs, imposing significant economic and social burdens [3, 9, 10].

The severity of CC varies considerably among individuals, ranging from mild discomfort to moderate or severe symptoms. A previous study revealed that constipation severity, duration, and associated symptoms result in a decline in quality of life [11]. Moreover, individuals with more severe CC often require more aggressive medical intervention due to the intensity of their symptoms and the risk of complications. As a result, greater severity is related to increased productivity losses and healthcare utilization. Therefore, determining the severity of CC, particularly its prevalence and risk factors, is critical.

In Asian countries such as China, Korea, Hong Kong, and India, the prevalence of CC has been reported to range from 8.2% to 16.8%, slightly lower than in Western populations [1, 12, 13, 14]. In Japan, the prevalence of CC varies widely, between 6.1% and 28.0%, depending on the diagnostic criteria applied [15, 16]. Among university students, CC is a common gastrointestinal disorder, with prevalence rates ranging from 5.1% to 16.2% across different settings and methodologies [17, 18]. This demographic represents a transitional phase between campus life and broader social interactions, as well as a critical period for physical and mental development. Unique vulnerabilities in this population, including lifestyle factors, dietary patterns, poor physical condition, and psychological distress, may contribute to gastrointestinal dysfunction, particularly CC [19]. Our recent study among Japanese university students reported a CC prevalence of 13.7% [20]. However, this investigation focused on the general student population without stratifying the severity of CC. There remains a worldwide gap regarding the prevalence and risk factors specific to severe CC compared with mild CC. This study aimed to identify the rate and risk factors associated with moderate‐to‐severe CC among Japanese university students, which may provide insights into its etiology and identify potential targets for intervention. These findings could also inform the development of management strategies for individuals suffering from CC.

## 2 Materials and Methods

### 2.1 Study Design and Participants

A cross‐sectional study was conducted to identify risk factors associated with moderate‐to‐severe CC among university students at Hiroshima University, a national institution located in Higashihiroshima and Hiroshima, Japan. The university comprises 12 undergraduate schools, including the School of Applied Biological Science, School of Integrated Arts and Sciences, School of Informatics and Data Science, School of Education, School of Economics, School of Engineering, School of Law, School of Letters, School of Science, School of Medicine, School of Dentistry, and School of Pharmaceutical Sciences. Additionally, there are five graduate schools: the Graduate School of Humanities and Social Sciences, the Graduate School of Advanced Science and Engineering, the Graduate School of Integrated Sciences for Life, the Graduate School of Biomedical and Health Sciences, and the Graduate School of Innovation and Practice for Smart Society.

Participants were recruited via an online reservation platform for annual health checkups in the academic year 2024 (from April 1 to June 30, 2024). All students at Hiroshima University who met the diagnostic criteria for CC per the ROME IV guidelines were eligible for inclusion. The exclusion criteria included participants who failed to provide informed consent, did not complete the survey, were pregnant, had neuromuscular disorders, or underwent long‐term treatment (≥ 2 weeks) with constipation‐inducing medications, including opioids and anticholinergic agents.

### 2.2 Definitions

CC was defined as the presence of at least two of the following symptoms persisting for three or more months, according to the Rome IV criteria [21]: 1) straining during more than 25% of defecations, 2) lumpy or hard stools in more than 25% of defecations, 3) sensation of incomplete evacuation during more than 25% of defecations, 4) sensation of anorectal obstruction or blockage during more than 25% of defecations, and 5) fewer than three spontaneous bowel movements per week.

The severity of CC was categorized into three levels. Mild CC was defined as either a Bristol Stool Form Scale (BSFC) score of 3 with SBM occurring twice per week or a BSFC score of 2 with SBM occurring three times per week and/or straining or a sensation of incomplete evacuation experienced during 25%–50% of defecations. Moderate CC was characterized by a BSFC score of 2, with SBM occurring twice per week, and/or straining or a sensation of incomplete evacuation occurring during 50%–75% of defecations. Severe CC was identified by a BSFC score of 1 and/or SBM occurring only once per week and/or straining or a sensation of incomplete evacuation during more than 75% of defecations. Participants were asked to report the severity of their constipation based on their experiences prior to initiating any pharmacological treatment or during periods when they were not receiving any constipation‐specific therapy.

Although instruments such as the Patient Assessment of Constipation‐Symptoms (PAC‐SYM) have been widely used in previous studies, this tool presents several limitations [22]. Specifically, PAC‐SYM is a relatively long and complex questionnaire, relies heavily on subjective symptom reporting, and does not explicitly capture objective measures such as the frequency of bowel movements per week, which is a core component of the Rome IV diagnostic criteria. Therefore, in this study, we developed the first pragmatic, symptom‐based categorization of CC severity, combining commonly used clinical indicators such as the BSFS, SBM, and symptom burden (e.g., straining, incomplete evacuation). These parameters were selected due to their frequent use in both research and clinical settings, as well as their relevance to the Rome IV diagnostic criteria.

Obesity was determined by a body mass index (BMI) of 25 kg/m^2^ or higher for the Asian population [23]. A family history of CC is defined as having at least one affected first‐degree relative. Participants who reported either abstinence from alcohol or consuming it no more than once per month were categorized as non‐drinkers. Smoking status was categorized as “never,” “former,” and “current” users. Physical inactivity was defined as the absence of any exercise lasting at least 20 min per day. Skipping breakfast was classified as not consuming any breakfast meals during the week. These lifestyle behaviors were assessed based on participants' routines over the previous six months. The Beck Depression Inventory (BDI) is a 21‐item self‐report instrument widely used to quantify depressive symptoms. Scores equal to or exceeding 30 on this scale were used as thresholds for identifying severe or extreme depression [24].

The Eating Attitudes Test‐26 (EAT‐26) is a validated tool designed to screen for disordered eating behaviors, with scores of 20 or higher suggesting the potential need for professional evaluation [25].

The Bulimic Investigatory Test (BITE), a 33‐item questionnaire, assesses binge‐eating tendencies, where scores of 20 or above indicate highly disordered eating patterns and the presence of binge eating [26].

### 2.3 Questionnaire Development

The survey questionnaire comprised three main sections. The first section addressed lifestyle factors, including smoking habits, alcohol use, physical activity, breakfast consumption, and average sleep duration. The second section focused on symptoms relevant to diagnosing CC and assessing its severity. The final section evaluated three psychological scales: the BDI, the Eating Attitudes Test (EAT)‐26, and the Bulimic Investigatory Test (BITE) [24, 25, 26]. The questionnaire included a variety of question formats, such as yes/no, multiple‐choice questions, and open‐ended responses.

A team of healthcare professionals from Hiroshima University's Health Service Center, consisting of three internists (SM, NI, and TH) and three psychologists (AY, YM, and YO), reviewed and refined the initial questionnaire. Following this revision, the questionnaire was piloted with 10 medical professionals to assess its validity. Feedback from the pilot study informed further modifications, and the questionnaire was subsequently translated into Japanese. To ensure accuracy and consistency, a reverse translation process was conducted. The translated version was pretested with 10 native Japanese speakers, and modifications were made as necessary based on the pretest results. The finalized English version of the survey questionnaire is provided in the S1 file.

### 2.4 Statistical Analysis

The obtained data were arranged into an Excel spreadsheet (Microsoft et al., USA) and analyzed with SPSS software version 23 (SPSS Inc., Chicago, IL). Descriptive statistics summarized baseline characteristics, whereas univariate analyses (chi‐square tests for categorical variables, t‐tests, or Mann–Whitney U tests for continuous variables) were conducted to identify potential associations. Multivariate logistic regression models were utilized to determine independent risk factors for moderate‐to‐severe CC, with the results reported as odds ratios (ORs) and 95% confidence intervals (CIs).

### 2.5 Ethical Considerations

Ethical approval for this study was obtained from the Ethical Committee of Hiroshima University, Japan (E‐143‐3). Informed consent was obtained from all participants before data collection and was included on the initial page of the online survey questionnaire. The survey questionnaires were collected fully anonymously and were not disclosed to any external organization. All methods were performed in accordance with the ethical standards of the institutional and national research committee and with the 1964 Helsinki Declaration and its later amendments.

## 3 Results

### 3.1 Participant Characteristics

**FIGURE 1:**
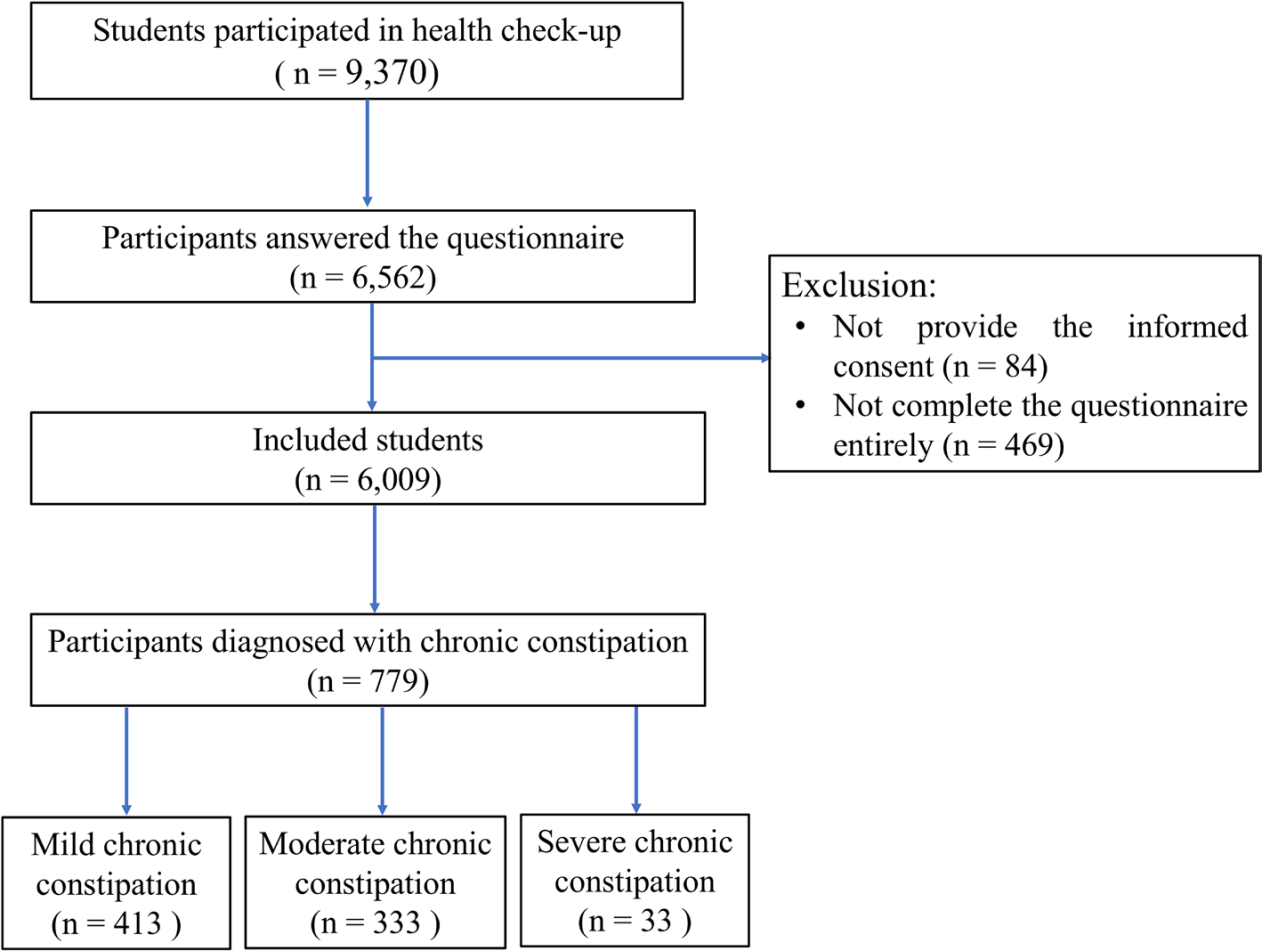
Flow chart of participant enrollment.

A total of 9370 students participated in the annual health checkup, of whom 6562 completed the web‐based questionnaire. Among them, 6009 responded to all survey questions. According to the Rome IV criteria, 779 students were diagnosed with CC, with 413 cases (53.0%) classified as mild CC, 333 cases (42.7%) as moderate CC, and 33 cases (4.2%) as severe CC (Figure 1).

In this study, the moderate and severe CC groups were combined for comparison with the mild CC group to emphasize the distinction between mild constipation and the more severe forms. This approach also ensured sufficient statistical power for analysis, as the number of severe cases was relatively small.

Table 1 presents the characteristics of the participants, who were divided into mild CC and moderate‐to‐severe CC groups. The mean age of the participants was 19.0 ± 3.1 years, with a male‐to‐female ratio of 1:1.96. Alcohol consumption was reported by 34.1% of the students, whereas 53.8% reported sleeping ≤ 6 h per day. With respect to breakfast habits, 14.6% of the students did not have breakfast. A total of 35.9% of the participants reported a history of CC among their first‐degree family members. In addition, 1.9% of the students had Eating Attitudes Test (EAT‐26) scores of 20 or higher, suggesting a potential need for professional evaluation of disordered eating behaviors.

**TABLE 1:** Characteristics of the participants.

Characteristics	Total (%) *N* = 779	Mild CC (%) *N* = 413	Moderate‐to‐severe CC (%) *N* = 366	*p*
Age of onset CC				
< 18 years	490 (62.9)	242 (58.6)	248 (67.8)	0.008
≥ 18 years	289 (37.1)	171 (41.4)	118 (32.2)	
Sex				
Male	263 (33.8)	160 (38.7)	103 (28.1)	0.002
Female	516 (66.2)	253 (61.3)	263 (71.9)	
Diabetes mellitus				
No	777 (99.7)	412 (99.8)	365 (99.7)	1
Yes	2 (0.3)	1 (0.2)	1 (0.3)	
Thyroid disease				
No	770 (98.8)	410 (99.3)	360 (98.4)	0.319
Yes	9 (1.2)	3 (0.7)	6 (1.6)	
Obesity				
No	732 (94.0)	390 (94.4)	342 (93.4)	0.563
Yes	47 (6.0)	23 (5.6)	24 (6.6)	
Alcohol consumption				
No	513 (65.9)	275 (66.6)	238 (65.0)	0.647
Yes	266 (34.1)	138 (33.4)	128 (35.0)	
Smoking				
No	751 (96.4)	400 (96.9)	351 (95.9)	0.477
Yes/ever	28 (3.6)	13 (3.1)	15 (4.1)	
Family history of CC				
No	499 (64.1)	283 (68.5)	216 (59.0)	0.006
Yes	280 (35.9)	130 (31.5)	150 (41.0)	
Physical exercises				
No	291 (37.4)	144 (34.9)	147 (40.2)	0.127
Yes	488 (62.6)	269 (65.1)	219 (59.8)	
Breakfast				
No	114 (14.6)	58 (14.0)	56 (15.3)	0.620
Yes	665 (85.4)	355 (86.0)	310 (84.7)	
Sleep duration				
≤ 6 h/day	419 (53.8)	208 (50.4)	211 (57.7)	0.042
> 6 h/day	360 (46.2)	205 (49.6)	155 (42.3)	
BDI				
< 30	758 (97.3)	402 (97.3)	356 (97.3)	0.953
≥ 30	21 (2.7)	11 (2.7)	10 (2.7)	
EAT‐26 score				
< 20	764 (98.1)	409 (99.0)	355 (97.0)	0.039
≥ 20	15 (1.9)	4 (1.0)	11 (3.0)	
BITE score				
< 20	757 (97.2)	404 (97.8)	353 (96.4)	0.248
≥ 20	22 (2.8)	9 (2.2)	13 (3.6)	

Abbreviations: BDI, Beck Depression Inventory; BITE, Bulimic Investigatory Test; CC, Chronic constipation; EAT‐26, Eating Attitudes Test‐26.

### 3.2 Risk Factors for Moderate‐To‐Severe CC

In the univariate analysis, age of CC onset < 18 years, female sex, family history of CC, sleep duration ≤ 6 h per day, and EAT‐26 score ≥ 20 were related to moderate‐to‐severe CC (Table 1). Table 2 displays the results of the multivariate logistic regression models. The factors significantly associated with moderate‐to‐severe CC in the multivariate model were age of CC onset < 18 years (odds ratio [OR]: 1.374; 95% CI [confidence interval]: 1.017–1.857; *p* = 0.039), female sex (OR: 1.444; 95% CI: 1.058–1.969; *p* = 0.02), family history of CC (OR: 1.449; 95% CI: 1.072–1.958; *p* = 0.016), and sleep duration ≤ 6 h per day (OR: 1.350, 95% CI: 1.011–1.802; *p* = 0.042).

**TABLE 2:** Univariate and multivariate logistic regression analyses of factors associated with moderate‐to‐severe CC.

Risk factors	Univariable			Multivariable		
	OR	95% CI	*p*	OR	95% CI	*p*
Age onset of CC < 18	1.485	1.107–1.992	0.008	1.374	1.017–1.857	**0.039**
Female	1.615	1.194–2.184	0.002	1.444	1.058–1.969	**0.020**
Family history of CC	1.512	1.127–2.029	0.006	1.449	1.072–1.958	**0.016**
No physical exercises	1.254	0.937–1.677	0.127	1.197	0.888–1.615	0.237
Short sleep duration (≤ 6 h per day)	1.342	1.011–1.781	0.042	1.350	1.011–1.802	**0.042**
EAT‐26 ≥ 20	3.168	1.000–10.038	0.039	3.114	0.965–10.042	0.057

### 3.3 Discussion

To the best of our knowledge, this is the first study evaluating the rate and risk factors for moderate‐to‐severe CC among Japanese university students. Our study revealed that the rate of moderate‐to‐severe CC was 47% among participants diagnosed with CC according to the ROME IV criteria. We also found that the age of CC onset < 18 years, female sex, family history of CC, and sleep duration ≤ 6 h per day were independent risk factors for moderate‐to‐severe CC compared with mild CC.

Our data revealed that the onset of CC before the age of 18 years was associated with moderate‐to‐severe CC; however, prior studies have not thoroughly examined this relationship. Wu et al. concluded that the early onset of CC in females is associated with increased symptom severity and pelvic floor dysfunction [27]. However, the age of onset in this study was higher, and the criteria used to define CC severity also differed from our study. Furthermore, Johanson et al. reported that symptom severity increased with the duration of constipation, with patients who experienced CC for six or more years reporting more severe symptoms than those with a duration of five years or less [11]. Nonetheless, the study did not examine the early onset of CC as a contributing factor. Additionally, children suffering from CC who receive insufficient care face a significantly increased risk of developing both physical and psychological issues, which may exacerbate burdens on already strained healthcare systems [28]. Consequently, early detection of constipation during adolescence and timely initiation of appropriate treatment are critical for mitigating disease severity. Additional prospective cohorts are needed to investigate the progression of CC from its onset to adulthood.

Previous studies revealed that females had a greater prevalence of CC than males did in the community, even among children, adults, and especially university students [1, 20, 29, 30]. This discrepancy can be explained by biological issues related to estrogen and progesterone, which cause slower gut transit [31]. Other factors include the role of the female pelvic floor in the biomechanics of gastrointestinal emptying [32]. Our study also revealed that, compared with males, females were associated with significantly more severe CC. Similarly, Mason et al. reported that women with idiopathic constipation have increased psychological and social morbidities, including anxiety, depression, social dysfunction, increased somatization, and decreased sexual satisfaction, which may contribute to poor quality of life [33]. In contrast, Bouchoucha et al. showed that constipation severity was not significantly associated with gender, although males tended to report lower severity than females [34]. Nevertheless, this study employed a Likert scale for constipation symptoms to assess constipation severity, which differed from the assessment method used in our study. Therefore, further studies should investigate the interaction between biological, psychological, and social factors to better elucidate the pathophysiology of severe constipation in females, from which personalized therapies should be designed.

Approximately 16.4% of constipated individuals had at least one first‐degree relative with constipation [35]. A positive family history of constipation and genetic predisposition appears to contribute to this illness since patients with functional constipation frequently report a positive family history [36]. Furthermore, similar dietary habits and lifestyle factors could also play a role in increasing the risk of CC within the family. Our results demonstrated that a first‐degree family history of CC was related to moderate‐to‐severe CC rather than mild CC. A previous study also indicated that CC patients with a positive family history experienced a longer duration of constipation, more complications, and more frequent use of digital evacuation [37]. Acknowledging a family history of constipation can help clinicians identify high‐risk groups and implement early interventions, such as health education, lifestyle modifications, and closer monitoring. This approach may reduce the severity of CC and prevent its long‐term complications.

Our previous research reported a correlation between reduced nighttime sleep duration and an increased prevalence of CC among university students, with those sleeping less than 6 h per night being at greater risk [20]. A recent meta‐analysis also indicated that insufficient sleep duration contributed to an increased risk of constipation [38]. In this study, sleep duration ≤ 6 h per day was an independent risk factor for moderate‐to‐severe CC compared with mild CC. Similarly, Yamamoto et al. reported that Japanese individuals with constipation and poor sleep quality experienced severe symptoms and poor quality of life [39]. Another study reported that insufficient sleep was related to worsened constipation symptoms, changes in anorectal function and perception, and impaired autonomic function in patients with CC [40]. A sleep duration of less than 6 h may disrupt the rapid eye movement (REM) sleep cycle [41], potentially leading to the development of gastrointestinal transit abnormalities [42]. Moreover, a sleep duration of 4–6 h or less could disrupt the circadian rhythm of gastrointestinal physiology, increasing nocturnal awakenings and resulting in abnormal colonic motility and contractions [43]. A short sleep duration might also increase susceptibility to constipation by influencing inflammatory markers such as interleukin‐6 and C‐reactive protein [44]. Furthermore, previous studies have demonstrated a close association between sleep deprivation and autonomic nervous system dysfunction [45]. Autonomic imbalance between the sympathetic and parasympathetic nervous systems, particularly increased sympathetic activity, impairs bowel motility and has been implicated in the pathophysiology of CC [46]. Therefore, health initiatives should aim at promoting adequate sleep hygiene among universities.

CC is a multifactorial disorder characterized by a complex pathogenesis that remains incompletely understood. In recent years, significant progress in metabolomics has created new opportunities to investigate the complex interactions between host health and gut microbiota. The gut microbiome composition of individuals with constipation significantly differs from that of healthy controls [47]. Gut dysbiosis may result in reduced production of short‐chain fatty acids (SCFAs), which are crucial for maintaining gastrointestinal function, thereby contributing to constipation. Besides the substantial impact of SCFAs on gut function, inflammatory, and immune responses, individuals with constipation also exhibit distinct metabolic features, including abnormal amino acid metabolism, changes in lipid metabolism, and gut microbial metabolites. Moreover, impaired bile acid metabolism, particularly a reduction in secondary bile acids such as deoxycholic acid, can interfere with receptor‐mediated signaling pathways and further impair intestinal motility. Imbalances in amino acid pathways and neurotransmitter synthesis may lead to neuromuscular dysfunction, while variations in microbial gas production, particularly shifts between methane and hydrogen, also influence gut transit dynamics [48]. Consequently, additional research is necessary to clarify the correlation between metabolomic profiles and the severity of CC.

This study has several limitations. First, the participants were exclusively drawn from a single national university in Japan, which may restrict the broader applicability of the findings. In addition, university students in Japan represent a unique subpopulation with distinct lifestyle characteristics, dietary patterns, and stress profiles that may not be generalizable to those in other parts of the world. Consequently, future research in diverse populations is necessary to validate our findings. Second, the data collection relied on a self‐administered, internet‐based survey, introducing the possibility of recall bias due to self‐reported information. The lack of clinical validation may result in misclassification of constipation severity. While this approach enabled broad participation and facilitated large‐scale data collection, it limits diagnostic accuracy and the clinical specificity of our findings. Additionally, selection bias may have occurred because participation was voluntary, and recruitment occurred through a health checkup platform. Students with greater health awareness may have been more likely to participate, potentially inflating prevalence estimates. Third, the severity classification used in our study was not derived from an established and validated scoring system, underscoring the necessity for future investigations to implement or establish standardized classifications for severity assessment in CC. Fourth, the cross‐sectional design of the study does not allow the establishment of causal relationships between CC severity and the identified risk factors. Fifth, we focused only on the effects of different sleep durations on constipation, with limited consideration given to sleep quality and other sleep‐related conditions. Furthermore, other potential influencing factors, such as dietary habits, existing comorbidities, and medication history, were not accounted for in this research. Finally, this study did not examine clinically relevant subgroups, such as individuals with irritable bowel syndrome with constipation, and did not collect data on abdominal pain, anorectal manometry findings, or defecatory disorders. However, due to the population‐based design and reliance on self‐reported data, clinical evaluations and physiological investigations were beyond the scope of this study. Future research should incorporate standardized diagnostic tools and clinical assessments to better characterize constipation subtypes and their underlying pathophysiology.

### 3.4 Conclusion

Our study revealed that the rate of moderate‐to‐severe CC was 47% among participants diagnosed with CC. Independent risk factors included female sex, younger age of onset, a family history of CC, and short sleep duration. These findings suggest that early diagnosis and development of management strategies may be needed to enhance the quality of life and health outcomes of affected individuals. Further research is warranted to confirm these associations using validated severity scales and to investigate the underlying mechanisms as well as potential prevention strategies to reduce the overall impact of moderate‐to‐severe CC.

## Ethics Statement

Ethical approval for this study was obtained from the Ethical Committee of Hiroshima University, Japan (E‐143‐3).

## Consent

Informed consent was obtained from all participants before data collection and was included on the initial page of the online survey questionnaire.

## Conflicts of Interest

The authors declare no conflicts of interest.
